# Adherence to a healthy lifestyle counteracts the negative effects of risk factors on all-cause mortality in the oldest-old

**DOI:** 10.18632/aging.102274

**Published:** 2019-09-16

**Authors:** Zhi Cao, Rui Wang, Yangyang Cheng, Hongxi Yang, Shu Li, Li Sun, Weili Xu, Yaogang Wang

**Affiliations:** 1 School of Public Health, Tianjin Medical University, Tianjin 300070, P.R. China; 2 Aging Research Center, Department of Neurobiology, Care Sciences and Society, Karolinska Institute and Stockholm University, Stockholm SE-17177, Sweden; 3 Department of Biostatistics, School of Public Health, Yale University, New Haven, CT 06520, USA

**Keywords:** risk factors, lifestyle, all-cause mortality, cohort study

## Abstract

In the study, we examined the extent to which the harmful effects of risk factors on all-cause mortality can be counteracted by healthy lifestyle practices in the oldest-old (80 years of age and older). A total of 17,660 oldest-old from China were followed up for up to 10 years. The data were analyzed using the Cox proportional hazard model with adjustment for potential confounders. We found that having a rural residence, not being married, having lower economic status, physical disability, impaired cognitive function, or comorbidity were all associated with an elevated risk of mortality. Using these factors, we computed a weighted “risk score.” Because never smoking, never drinking, doing physical exercise, having an ideal diet, and a normal weight were independently associated with lower mortality, we also combined them to compute a weighted “protection score.” Both scores were divided into lowest, middle, and highest groups using their tertiles. In joint effect analyses, participants with the combined highest-risk score and lowest-protection score profile had a nearly threefold higher joint death risk. These analyses show that adherence to a healthy lifestyle counteracts the negative effect of risk factors on all-cause mortality in the oldest-old by more than 20%.

## INTRODUCTION

Worldwide populations are aging rapidly due to increased life expectancy and reduced mortality in late-life [1–4]. More than 23 million oldest-old individuals (over 80 years of age) live in China, contributing 18% of the oldest-old population worldwide in 2015 [5]. As the Chinese population ages over the coming decades, this percentage is expected to rapidly grow. By 2050, over a quarter of the global oldest-old population will live in China [6]. Population aging in China will bring dramatic family and economic burdens to Chinese society, with the medical care system being particularly overburdened.

Annual mortality among oldest-old individuals was reduced by somewhere between 0.2% and 1.3% from 1998 to 2008 in China [7]. Impaired cognitive functions were independent predictors of all-cause mortality in very old people [8]. Moreover, the risk of mortality is very high for the oldest-old with disabilities [9]. Additionally, socioeconomic inequalities, obesity, cardiovascular factors, and chronic diseases are associated with mortality in the oldest-old [10–13]. Conversely, healthy lifestyle practices, such as consumption of fruits and vegetables, social participation, and maintaining a normal weight, are associated with lower mortality [14–16]. The question remains as to whether a healthy lifestyle and behavioral factors (*e.g.,* never smoking and physical training) can somehow compensate for the harmful effects of the risk factors on mortality.

Given the large number of adults aged 80 years and older, and the increasing emphasis on managing the aging process, it is important to understand what are the determinants of healthy longevity, and whether certain lifestyles can counteract the harmful effect of risk factors on mortality among oldest-old. In the present study, therefore, we aimed to 1) identify independent risk factors and investigate the combined effect of risk factors and healthy lifestyle on all-cause mortality, and 2) explore the extent to which the negative effect of risk factors on mortality can be counteracted by healthy lifestyle factors.

## RESULTS

### Characteristics of study population

The 17660 participants (mean age 92.7 years) included 10758 women and 6902 men. During the follow-up, 11094 (62.8%) in total, and 4298 (62.3%) men and 6796 (63.2%) women died. Table 1 shows the characteristics of study population by gender. Compared with males, females were older, more likely not to be married, to be illiterate and underweight, and to live in a rural residence, to have a lower economic level, a lower systolic and diastolic blood pressure (BP), a higher heart rate, a physical disability, impaired cognitive function, an unhealthy diet, and less comorbidity and physical exercise, and tended to be non-smoker and non-drinker.

**Table 1:** Baseline characteristics of the study population by gender.

**Characteristics**	**Total (N=17660)**	**Male (N=6902)**	**Female (N=10758)**	***P* value**
**Sociodemographic factors**				
**Age** (years), Mean (SD)	92.7 (7.0)	90.6 (6.5)	94.1 (7.1)	<0.001
**Ethnic**				0.362
Han	16572 (93.8)	6491 (94.0)	10081 (93.7)	
Non-Han	1088 (6.2)	411 (6.0)	677 (6.3)	
**Marital status**				<0.001
In marriage	2988 (16.9)	2272 (32.9)	716 (6.7)	
Not in marriage	14672 (83.1)	4630 (67.1)	10042 (93.3)	
**Education level**				<0.001
Literate	4645 (26.3)	3564 (51.6)	1081 (10.0)	
Illiterate	13015 (73.7)	3338 (48.4)	9677 (90.0)	
**Residence**				<0.001
Urban	7229 (40.9)	2957 (42.8)	4272 (39.7)	
Rural	10431 (59.1)	3945 (57.2)	6486 (60.3)	
**Economic level**				0.010
Low	11177 (63.3)	4282 (62.0)	6895 (64.1)	
Middle	4586 (26.0)	1831 (26.5)	2755 (25.6)	
High	1897 (10.7)	789 (11.4)	1108 (10.3)	
**Cardiovascular profiles**				
**Systolic BP (mmHg)**				<0.001
<110	1241 (7.0)	421 (6.1)	820 (7.6)	
110–139	10154 (57.5)	4039 (58.5)	6115 (56.8)	
140–159	4165 (23.6)	1655 (24.0)	2510 (23.3)	
≥160	2100 (11.9)	787 (11.4)	1313 (12.2)	
**Diastolic BP (mmHg)**				0.003
<70	2320 (13.1)	841 (12.2)	1479 (13.7)	
70-89	11077 (62.7)	4332 (62.8)	6745 (62.7)	
≥90	4263 (24.1)	1729 (25.1)	2534 (23.6)	
**Heart rate**				<0.001
<60	678 (3.8)	283 (4.1)	395 (3.7)	
60–79	12834 (72.7)	5106 (74.0)	7728 (71.8)	
≥80	4148 (23.5)	1513 (21.9)	2635 (24.5)	
**Health status**				
**Physical function**				<0.001
Normal	12202 (69.1)	5325 (77.2)	6877 (63.9)	
Disability	5458 (30.9)	1577 (22.8)	3881 (36.1)	
**Cognitive function**				<0.001
Normal	5200 (29.4)	2875 (41.7)	2325 (21.6)	
Mild	5266 (29.8)	2145 (31.1)	3121 (29.0)	
Moderate	3146 (17.8)	886 (12.8)	2260 (21.0)	
Severe	4048 (22.9)	996 (14.4)	3052 (28.4)	
**Comorbidity**				<0.001
=0	13867 (78.5)	5225 (75.7)	8642 (80.3)	
≥1	3793 (21.5)	1677 (24.3)	2116 (19.7)	
**Lifestyle factors**				
**Smoking**				<0.001
Never	12591 (71.3)	3118 (45.2)	9473 (88.1)	
Former	2628 (14.9)	1927 (27.9)	701 (6.5)	
Current	2441 (13.8)	1857 (26.9)	584 (5.4)	
**Drinking**				<0.001
Never	12719 (72.0)	3630 (52.6)	9089 (84.5)	
Former	2161 (12.2)	1454 (21.1)	707 (6.6)	
Current	2780 (15.7)	1818 (26.3)	962 (8.9)	
**Physical exercise**				<0.001
Never	11559 (65.5)	3871 (56.1)	7688 (71.5)	
Former	1642 (9.3)	661 (9.6)	981 (9.1)	
Current	4459 (25.2)	2370 (34.3)	2089 (19.4)	
**Diet**				<0.001
Nonideal diet	4810 (27.2)	1812 (26.3)	2998 (27.9)	
Ideal diet	12850 (72.8)	5090 (73.7)	7760 (72.1)	
**BMI (kg/m^2^)**				<0.001
<18.5	8364 (47.4)	2693 (39.0)	5671 (52.7)	
18.5-24.9	8457 (47.9)	3829 (55.5)	4628 (43.0)	
≥ 25	839 (4.8)	380 (5.5)	459 (4.3)	

Abbreviations: SD: standard deviation; BP: blood pressure; BMI: body mass index.

### Risk factors, healthy lifestyle and mortality

**Figure 1:**
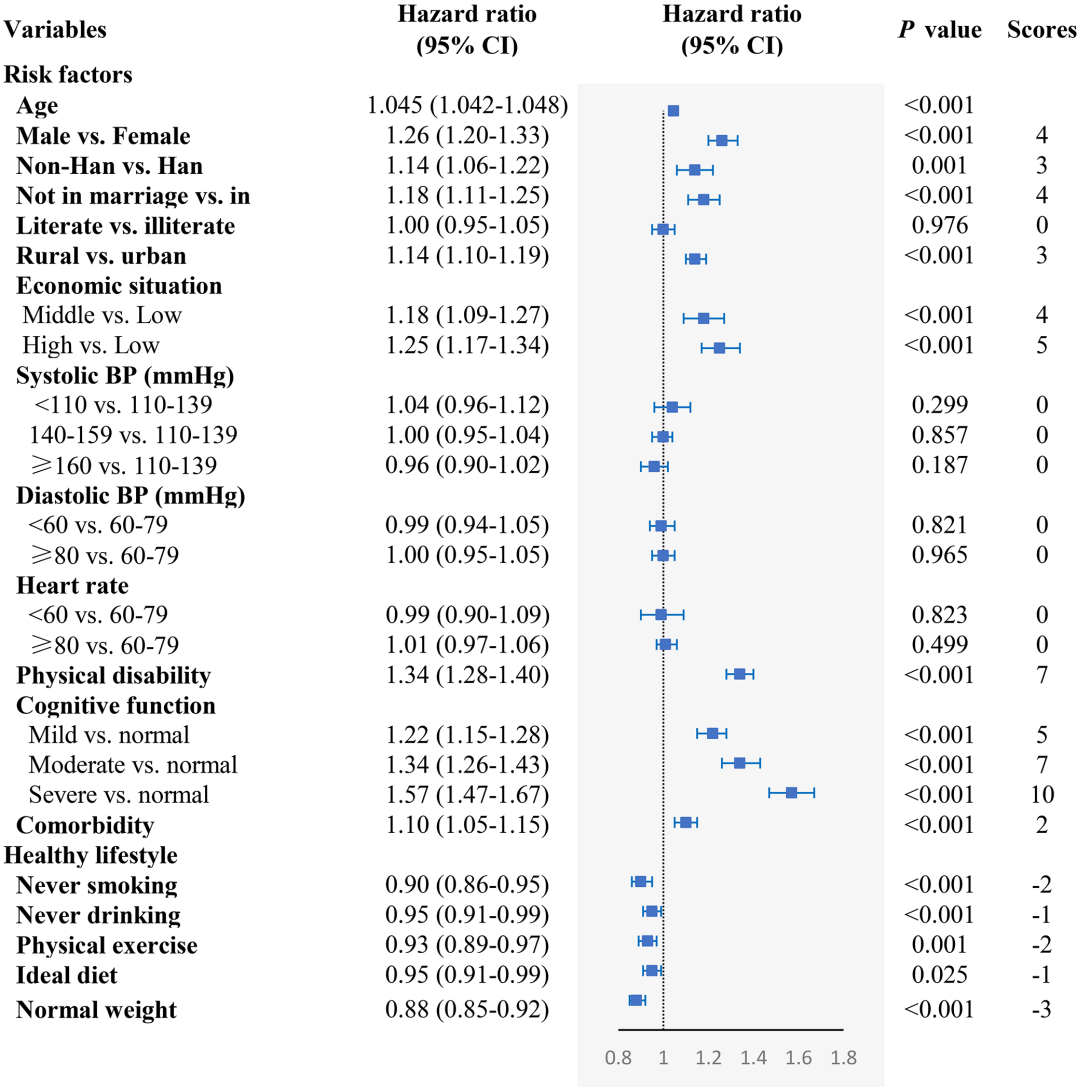
**The association of the risk of mortality in relation to demographic characteristics, cardiovascular profile, health condition, and healthy lifestyle.** The model included age, gender, residence, ethnicity, education level, economic situation, marital status, systolic BP, diastolic BP, heart rate, physical disability, cognitive function, comorbidity, smoking status, drinking, exercising, diet, and BMI, which were adjusted for each other.

We found that rural residence, not in marriage, lower economic situation, physical disability, impaired cognitive function, and comorbidity were independently associated with higher mortality. On the other hand, never smoking, never drinking, physical exercise, ideal diet, and normal weight were associated with lower mortality (Figure 1). In assessing the adverse effect of the five unhealthy lifestyles on mortality, we found that former and current smoking, former drinking, never exercising, and underweight were associated with increased mortality, while there was no significant association between current drinking and overweight with mortality (Supplementary Table 1). Moreover, systolic BP and diastolic BP were not associated with mortality, neither in males nor in females (*P* > 0.05) (Supplementary Table 2). When taking into account the effect of adverse health conditions on mortality, we excluded the oldest-old with impaired cognition, disability and comorbidity; nonetheless, we found similar independent risk factors related to mortality for all other adverse health conditions considered (Supplementary Table 3).

### Joint effect of risk factors and healthy lifestyle on mortality

We generated a composite “weighted risk score” of mortality by gender, ethnicity, residence, marital status, economic situation, physical activity, cognitive health, and comorbidity together, ranging from 0 to 39. In contrast, a “weighted protection score” against mortality was composed of healthy factors such as never smoking, never drinking, physical exercise, ideal diet, and normal weight, with the range being 0–9.

Cox models analyses showed that our risk score correlated positively with risk of mortality (HR: 1.065, 95% CI: 1.061–1.068) while our protection score was inversely correlated with mortality (HR: 0.94, 95% CI: 0.93–0.95) (Table 2). We divided the risk and protection scores of all participants into three groups according to their tertiles. Compared with the lowest tertile, the HR of mortality for the oldest-old in the high and middle risk score groups was 2.38 (2.27–2.50) and 1.46 (1.38–1.53), respectively. On the other hand, the HR of mortality for people in the high and middle protection score groups was 0.74 (0.70–0.77) and 0.91 (0.87–0.96), respectively.

**Table 2:** The association of independent risk factor score and healthy lifestyle score with mortality.

	**Total**		**Male**		**Female**	
		**HR (95% CI)**	***P* value**	**HR (95% CI)**	***P* value**	**HR (95% CI)**	***P* value**
**Risk factors**						
Risk score, continuous	1.065 (1.061–1.068)	<0.001	1.060 (1.055–1.065)	<0.001	1.069 (1.065–1.073)	<0.001
Tertile of risk score						
Lowest	1 (Ref.)		1 (Ref.)		1 (Ref.)	
Middle	1.46 (1.38–1.53)	<0.001	1.58 (1.48–1.70)	<0.001	1.48 (1.39–1.58)	<0.001
Highest	2.38 (2.27–2.50)	<0.001	2.30 (2.13–2.48)	<0.001	2.55 (2.40–2.71)	<0.001
**Healthy lifestyles**						
Protecting score, continuous	0.94 (0.93–0.95)	<0.001	0.94 (0.93–0.95)	<0.001	0.93 (0.92–0.94)	<0.001
Tertile of protecting score						
Lowest	1 (Ref.)		1 (Ref.)		1 (Ref.)	
Middle	0.91 (0.87–0.96)	<0.001	0.92 (0.85–0.99)	0.025	0.89 (0.84–0.95)	0.001
Highest	0.74 (0.70–0.77)	<0.001	0.74 (0.69–0.79)	<0.001	0.72 (0.68–0.77)	<0.001

**Figure 2:**
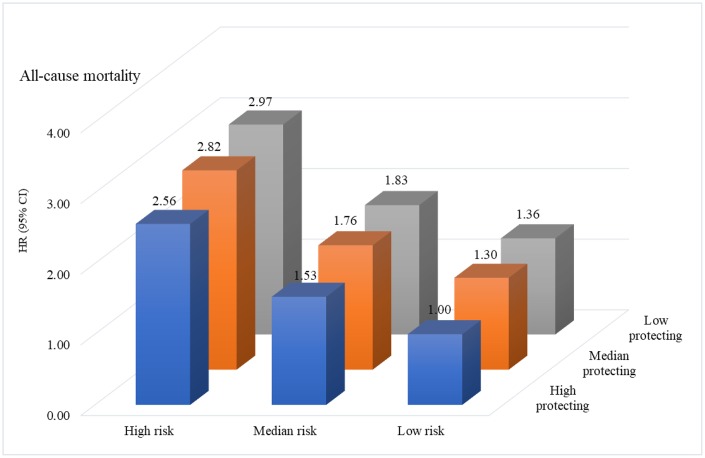
**The joint effect of risk factors and healthy lifestyle scores on risk of mortality.**

In assessing the joint effect of risk factors and healthy lifestyles on mortality, we found that participants in the combined highest risk score and lowest protection score profile had a nearly threefold risk of mortality compared with the lowest risk and the highest protecting score profile. In the highest risk score profile, the risk of mortality obviously increased with decreases in protection score (high score: HR=2.97, 2.76–3.20; middle: HR=2.82, 2.62–3.03; low score: HR=2.56, 2.38–2.75) (Figure 2, Supplementary Table 4). The trend of increasing risk of mortality with decreasing protection scores was consistent with median and low risk score profiles. Furthermore, the association of risk score profile with mortality differed when stratified by protection score profile (*P* for interaction = 0.018).

### The harmful effect of risk factors on mortality was counteracted by healthy lifestyle

**Figure 3:**
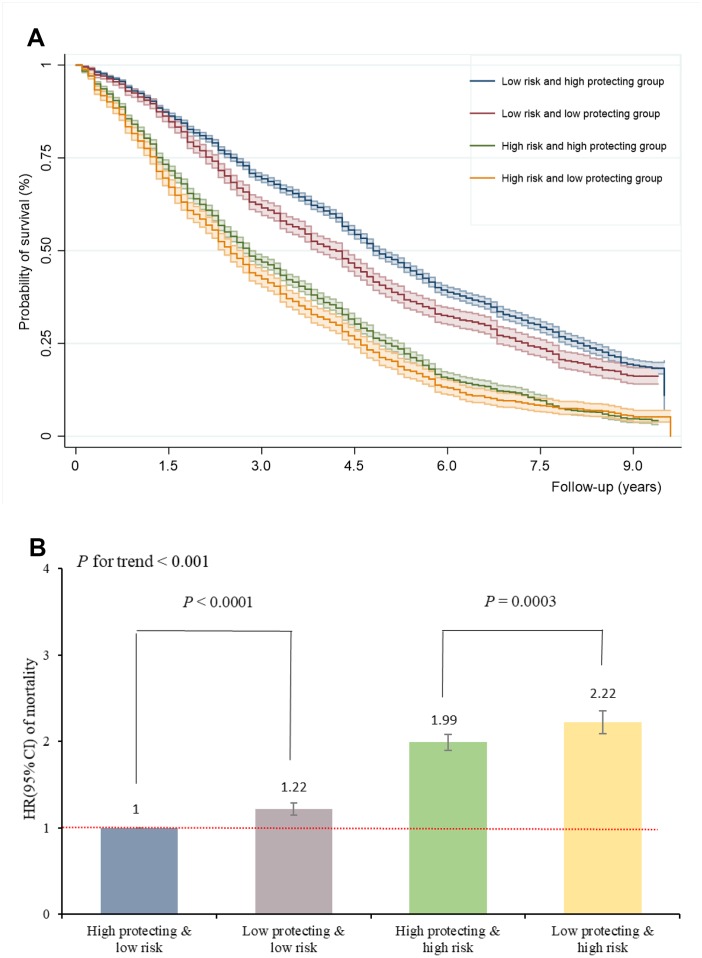
**Adherence to healthy lifestyle can counteract the harmful effect of risk factors on mortality and prolong survival in oldest-old.** (**A**) The Kaplan-Meier survival curves of combined risk and protecting factors; (**B**) The HRs of combined risk and protecting factors for mortality.

Kaplan-Meier survival analysis showed that the median survival time in the joint groups of low risk and high protection, low risk and low protection, high risk and high protection, high risk and low protection, was 4.8, 4.2, 2.8 and 2.5 years, respectively (*P* for log-rank < 0.001), suggesting that high protection scores may prolong survival in the oldest-old with high risk scores (Figure 3A). Figure 3B shows the offsetting effect of adherence to a healthy lifestyle on the association between risk factors and the risk of mortality. In the low risk profile, high protection scores can further reduce the risk of mortality (*P* < 0.0001). Similarly, mortality was strongly attenuated by high protection scores among the high-risk profile (*P* = 0.0003). High (middle and highest) protection scores counteracted the negative effect of high (middle and highest) risk scores on mortality by 23%. The proportion of incident death cases that was attributable to a high protection score profile was 0.26 (95% CI 0.24-0.27) among participants with a low risk profile, and 0.47 (95% CI 0.43-0.52) among those with a high risk factor profile. These findings suggest that death among the oldest-old with a high risk profile might be preventable in 26% of cases by having a healthy lifestyle (and thus a high protection score), implying not smoking, not drinking, ideal diet, exercising, and normal weight. Moreover, for the oldest-old with high risk profiles, having a high-protection score can prevent death in 47% of cases.

## DISCUSSION

In this large, nationwide cohort study of Chinese oldest-old (80 years of age and older), we found that rural residence, not in marriage, lower economic level, physical disability, impaired cognitive function, and comorbidity are independent risk factors. On the other hand, we also found that never smoking, never drinking, physical exercise, ideal diet, and normal weight are protective factors for all-cause mortality among our cohort. Participants in the combined highest risk score profile and lowest protection score profile had a nearly threefold risk of mortality compared with the combined lowest risk and the highest protection scores profile. Importantly, adherence to a healthy lifestyle might counteract the harmful effects of independent risk factors on risk of mortality by 23%.

Based data from ~0.5 million participants in the UK biobank cohort, Bhautesh *et al.* [17] found that socioeconomic status was a predictor of all-cause mortality while mortality risk with an increasing number of long-term health conditions remained constant across different socioeconomic gradients. In our study, ethnicity, residence, marital status, and economic level were independently associated with death risk. The latter factor suggested that a higher socioeconomic status, which allows easier availability to healthcare and healthy lifestyle practices, might reduce the risk of mortality in the oldest-old. It’s important to emphasize that cardiovascular factors, including systolic and diastolic BP as well as heart rate, were not statistically associated with death risk in our study. Although these results are consistent with a previous study targeting very old people [11, 18–21], and with studies that did not find any association between BP and mortality in 80-year-old subjects in Japanese [19] and American/Western European [18, 20] cohorts, controversy remains regarding the association between BP and mortality in old age. Lv *et al.* [22] revisited a U-shaped association between systolic BP and mortality based on a total of 4,658 oldest-old assessed at three years of follow-up, finding no obvious association of diastolic BP, mean arterial pressure, and pulse pressure with all-cause mortality. We do not know the precise mechanism responsible for this discrepancy. Consequently, the U-shaped association between systolic BP and mortality needs to be further verified targeting very old people in future studies. In addition, we demonstrated that underweight, rather than overweight, was associated with increased risk of mortality among the oldest-old, which was consistent with previous study [15, 23]. These data suggested that current international and national recommendations focusing on the risks of excess body mass index (BMI) in the oldest-old may need to be revisited, and more attention should be paid in estimating the death risk of lower BMI.

In line with our findings, Lv *et al.* [24] recently found faster cognitive decline was associated with higher mortality independent of initial cognitive function, especially among those aged 65–79 years. Similar results, namely that poor cognitive performance is an independent risk factor for mortality, were also shown in participants aged 48–92 years in Europe [25]. Mutambudzi *et al.* [26] observed an association between both low-declining and high-declining trajectories of physical performance and increased risk of mortality in Mexican Americans aged 75–109 years. Similarly, our study indicated that disability in daily-living activities contributed to mortality both in men and women among the oldest-old.

Our study suggested that adherence to a healthy lifestyle may reduce the risk of morbidity and mortality by counteracting the harmful effect of independent risk factors on risk of mortality among the oldest-old. Zhang *et al.* [27] reported that the oldest-old with more negative self-perception of aging tended to engage in more healthy lifestyles (*e.g.*, eating fresh vegetables and fruits, exercising regularly, and not smoking), which could lead to a decreased risk of mortality. Adherence to a healthy lifestyle, as a potential modifier, can compensate for the harmful effect of death risk factors. With respect to previous studies done in this field, it is worth noting that an active and socially integrated lifestyle (mental, social, and physical leisure activities, and having a rich social network) may significantly counteract the detrimental effect of diabetes on dementia risk in older people [28]. Loes *et al.* [29] evaluated the associations of a polygenic risk score and healthy lifestyle with incident stroke using the UK biobank dataset, and found that genetic and lifestyle factors were independently associated with risk of incident stroke, highlighting the potential of lifestyle interventions to reduce risk of stroke across entire populations, even in those at high genetic risk. Similarly, genetic composition and combined health behaviors and factors had a log-additive effect on the risk of developing cardiovascular disease; consequently, behavioral lifestyle changes should be encouraged for all through comprehensive, multifactorial approaches [30]. Despite the benefits that could result from the entire population adhering to healthy lifestyle practices, to our knowledge, this is the first study to determine the offsetting effect of a healthy lifestyle on the association between independent risk factors and increased risk of mortality in adults aged over 80 years.

### Strengths and limitations

Our study has several strengths. It is a longitudinal study based on a very old population of China, and participants were recruited from a large general population, which allows for some level of generalization of our results. Furthermore, we tried to include multiple types of potential influence factors to counterbalance each other, allowing to examine the effect of independent risk factors and the benefits of healthy lifestyle practices on mortality more precisely. Despite the strengths of the study, there are several limitations. Firstly, although we identified many independent risk factors of all-cause mortality in the oldest-old in terms of demographic characteristics, health conditions, cardiovascular factors, and lifestyle, many other factors are not fully taken into consideration in this study. Secondly, people who survive to age 80 could already represent a group of mostly healthy individuals; indeed, the number of participants with comorbidity ≥ 2 was only 3.6% of our cohort. Therefore, we calculated a composite comorbidity score for self-reported major disease of the oldest-old. Thirdly, due to limited information, we did not include quantified alcohol intake or exercise intensity; rather, we divided participants into three groups in terms of drinking current drinkers, former drinkers, and never drinkers, depending on the time of alcohol consumption through their lifetime.

## CONCLUSIONS

In summary, in this nationwide, community based prospective study among the Chinese oldest-old, risk and protective factors in relation to mortality were identified. We found that the harmful effect of risk factors on mortality could be counteracted by adherence to a healthy lifestyle in the oldest-old. Our findings further confirm the efficacy of an integrated approach to healthy longevity, which considers various lifestyle practices in conjunction (as opposed to in isolation) among the oldest-old. Our results emphasize the value of promoting healthy living as a preventive strategy, and to improve the management of healthy lifestyles. From a public health perspective, future studies with life course data could help clarify how environmental factors and behaviors interact with genetic and epigenetic factors to influence death risk.

## MATERIALS AND METHODS

### Study population

This study draws on data from the oldest-old participants (aged over 80 years) from the 2005 and 2014 waves of the Chinese Longitudinal Healthy Longevity Surveys (CLHLS). The CLHLS is a nationwide survey done in a randomly-selected half of the counties and cities in 22 of the 31 provinces in China. A multistage cluster sampling approach was used in this prospective, longitudinal, community-based study. The CLHLS attempted to interview all centenarians who voluntarily agreed to participate in the study in the sampled counties and cities. Details of the sampling procedure and descriptions of CLHLS are available elsewhere [7, 15, 22]. All participants were interviewed about demographic characteristics, medical history, lifestyle, and health behaviors, and physical examinations with a standardized questionnaire and relevant instruments after obtaining informed consent from them. For the current study, we used data over a 10-year period with four assessments. The participants were initially recruited in 2005 (N=15,638), 2008 (N=9,479) and 2011 (N=1,360), and followed up until 2014. Excluding individuals under 80 or above 105 years of age (N=7,630), inaccurate date of death (N=140), and missing data (N=1,047), a total of 17,660 participants (aged 80-105 years) were eligible for the study.

### Data collection

Data on demographic characteristics (age, gender, residence, marital status, education level, economic situation), health conditions (cognitive function, physical disability, comorbidity), cardiovascular factors (systolic BP diastolic BP, heart rate), and lifestyle (smoking, drinking, diet, physical exercise, BMI) were collected through structural interviews and physical measurement. Given the high illiteracy rate, auditory and visual impairments, and other inconveniences (especially among centenarians), some of the oldest-old agreed to participate in the study through proxy assistance by a close family member. No proxy was used for objective questions such as assessment of cognitive function and physical disability.

Cognitive function was assessed by the Mini-Mental State Examination (MMSE) [31], which has been widely applied in epidemiological studies. We classified participants in different cognitive function categories depending on their MMSE scores (which ranged from 0 to 30 points), as follows: no cognitive impairment (25-30), mild cognitive impairment (18-24), moderate cognitive impairment (10-17), and severe cognitive impairment (0-9) [32]. Physical disability was identified by Activities of Daily Living (ADL), which was measured by the participant’s self-reported results in six self-care tasks consisting of bathing, dressing, eating, indoor transferring, toileting, and continence based on the Katz index scale [33]. Physical disability was defined as a need for assistance or a difficulty in one or more of the six activities listed above [34]. Furthermore, a healthy diet was determined by the consumption of fruit, vegetables, and fish and abstinence from meats. We defined a healthy diet as adherence to at least two of the healthy food items listed above [29]. According to the self-reported chronic disease status of participants, we computed a comorbidity score taking into account instances of diabetes, heart disease, stroke, asthma, and cancer (categorized as ≥1 and =0), diagnose by a specialist doctor.

### Identification of death

Mortality status was ascertained during the follow-up survey in 2014, assessing whether subjects died and the date of death, completed the study, or were lost to follow-up. Information about death was ascertained and affirmed by a close family member or village doctor.

### Statistical analyses

We summarized the participants’ baseline characteristics using descriptive statistics, reporting the mean and standard deviation (SD) of normal distribution or median and interquartile ranges of non-normal distribution for continuous variables, and proportions for categorical variables. We compared the baseline characteristics by all-cause mortality using chi-square test for categorical or student *t* test for continuous variables.

We calculated the follow-up time from the date of enrollment to the date of death, or loss to follow-up, or the end of the follow-up period, whichever came first. A “lost to follow-up” status was designated to those who could not be found and contacted. Participants who survived or were lost to follow-up were censored in 2014. The Cox proportional hazards regression model was used to calculate the hazard ratios (HRs) and 95% confidence intervals (CIs) of mortality in relation to risk factors and healthy lifestyle. Schoenfeld residuals were used to assess whether proportionality assumptions were satisfied, the results of which suggested that the assumptions were not violated. Age, gender, residence, ethnicity, education level, economic situation, marital status, systolic BP, diastolic BP, heart rate, physical disability, cognitive function, comorbidity, smoking status, drinking, exercising, diet, and BMI were adjusted for each other. We also did sensitivity analyses for which the oldest-old with impaired cognitive function, disability, and comorbidity were excluded to avoid the adverse causality of worsening health conditions on mortality.

According to the effects of each factor on all-cause mortality, we classified gender, ethnicity, residence, poor economic situation, unmarried status, physical disability, cognitive function, and comorbidity as independent risk factors for mortality, while never smoking, never drinking, physical exercise, ideal diet, and normal weight were classified as healthy lifestyle practices that decreased the risk of mortality. Then we created two composite scores to investigate the joint effect of independent risk factors and healthy lifestyle practices on mortality. The composite score was calculated in three steps [35, 36], as follows: 1) run a multivariable Cox regression model that includes all potential risk factors and healthy lifestyle practices to estimate the effect of each factor independent of potential confounders; 2) calculate the individual risk point for each response category of each variable by dividing the respective regression coefficient (β coefficient) with a single constant, which represents the regression coefficient for a one year increase in age in relation to risk of mortality; 3) round the risk points to the nearest integers and calculate the composite score by summing them up (Supplementary Table 5). Furthermore, independent risk factors and healthy lifestyle scores were treated as both continuous variables and categorical variables (tertiles) in the Cox regression model to test the dose-response relationship of risk/lifestyle factors with mortality. We also tested the statistical interactions between risk factors and healthy lifestyle scores in relation to mortality in the Cox model.

To further investigate whether and to what extent healthy lifestyle practices can counteract the harmful effect of the independent risk factors on mortality [28], we created a variable with four categories, which combines the tertiles of risk factor score (high level: top tertile vs low level: middle/bottom tertiles) with the tertiles of the healthy lifestyle score (high level: middle/top tertiles vs low level: bottom tertiles). This combined variable divided participants into four groups according to their risk and healthy lifestyle profiles: 1) low risk & high protection scores; 2) low risk & low protection scores; 3) high risk & high protection scores; (4) high risk & low protection scores. To quantify the contribution of joint risk factors and healthy lifestyle to mortality, we calculated the population attributable fraction (PAF), which is the estimated proportional reduction in mortality that would occur if risk factors and unhealthy lifestyle practices were prevented. We used STATA 15.0 for all statistical analyses. All statistical tests were two sided, and we considered *P*<0.05 to determine statistical significance.

## Supplementary Material

Supplementary Tables
