# Supplementary material for: Adherence to a healthy lifestyle counteracts the negative effects of risk factors on all-cause mortality in the oldest-old
Source: Aging (Albany NY). 2019 Sep 16;11(18):7605–19. doi: 10.18632/aging.102274 (PMC6781993; doi:10.18632/aging.102274)
Supplement: Supplementary Tables [file aging-11-102274-s001.pdf]

SUPPLEMENTARY TABLES

Supplementary Table 1. The adverse effect of unhealthy lifestyles on risk of mortality among oldest-old.

Lifestyles	HR (95% CI)	*P* value
**Smoking**		
Never	1 (Ref.)	
Former smoking	1.14 (1.08–1.21)	<0.001
Current smoking	1.07 (1.01–1.14)	0.027
**Drinking**		
Never	1 (Ref.)	
Former drinking	1.11 (1.05–1.18)	0.001
Current drinking	1.01 (0.96–1.07)	0.619
**Exercising**		
Current exercising	1 (Ref.)	
Former exercising	1.04 (0.96–1.12)	0.318
Never exercising	1.09 (1.04–1.14)	0.001
**Diet**		
Nonideal diet	1.05 (1.01–1.09)	0.032
**BMI**		
Normal weight (18.5–24.9 kg/m^2)	1 (Ref.)	
Underweight (<18.5 kg/m^2)	1.14 (1.10–1.19)	<0.001
Overweight (≥ 25 kg/m^2)	1.02 (0.93–1.12)	0.640

Model was adjusted for age, gender, residence, ethnic, education level, economic level, marital status, systolic BP, diastolic BP, heart rate, physical disability, cognitive function, comorbidity.

Supplementary Table 2. The gender difference of association between BP and mortality in oldest-old.

Blood pressure (BP)	Male		Female		*P* for interaction
	HR (95% CI)	*P* value	HR (95% CI)	*P* value	
**Systolic BP (mmHg)**					0.331
<110 vs. 110–139	1.05 (0.92–1.20)	0.473	1.04 (0.95–1.14)	0.426	
140–159 vs. 110–139	1.05 (0.97–1.13)	0.206	0.96 (0.91–1.02)	0.225	
≥160 vs. 110–139	0.97 (0.87–1.07)	0.502	0.95 (0.88–1.03)	0.232	
**Diastolic BP (mmHg)**					0.955
<60 vs. 60–79	1.01 (0.91–1.11)	0.919	0.98 (0.91–1.06)	0.678	
≥80 vs. 60–79	0.98 (0.91–1.05)	0.574	1.01 (0.95–1.08)	0.650	

Model was adjusted for age, residence, ethnic, education level, economic level, marital status, heart rate, physical disability, cognitive function, comorbidity, smoking, diet, drinking, physical exercising, BMI, as well as systolic BP or diastolic BP.

Supplementary Table 3. The association of the risk of mortality in relation to sociodemographic factors, cardiovascular profile, health status, and lifestyle among normal cognition, physical performance and free of comorbidity.

Variables	Oldest-old without impaired cognition	Oldest-old without physical disability	Oldest-old without comorbidity
**Age**	1.045 (1.041–1.049)	1.056 (1.052–1.060)	1.049 (1.046–1.053)
**Male vs. Female**	1.21 (1.14–1.28)	1.35 (1.27–1.43)	1.25 (1.18–1.33)
**Non-Han vs. Han**	1.16 (1.06–1.27)	1.08 (0.99–1.17)	1.17 (1.08–1.27)
**Not in marriage vs. In marriage**	1.11 (1.03–1.19)	1.25 (1.17–1.34)	1.19 (1.11–1.27)
**Literate vs. illiterate**	0.97 (0.91–1.03)	1.06 (0.99–1.12)	1.02 (0.96–1.08)
**Rural vs. Urban**	1.13 (1.08–1.19)	1.13 (1.07–1.19)	1.13 (1.08–1.19)
**Economic level**			
Middle vs. Low	1.26 (1.15–1.37)	1.09 (0.99–1.20)	1.18 (1.08–1.29)
High vs. Low	1.35 (1.25–1.47)	1.15 (1.05–1.25)	1.23 (1.14–1.33)
**Systolic BP (mmHg)**			
<110 vs. 110–139	1.08 (1.00–1.18)	1.07 (0.98–1.18)	1.03 (0.94–1.12)
140–159 vs. 110–139	0.99 (0.94–1.05)	0.99 (0.93–1.05)	0.99 (0.94–1.04)
≥160 vs. 110–139	0.99 (0.92–1.07)	0.92 (0.85–1.00)	0.95 (0.89–1.02)
**Diastolic BP (mmHg)**			
<60 vs. 60–79	0.99 (0.93–1.06)	0.98 (0.91–1.05)	1.00 (0.94–1.07)
≥80 vs. 60–79	0.97 (0.93–1.03)	1.00 (0.95–1.06)	1.00 (0.95–1.06)
**Heart rate**			
<60 vs. 60–79	1.03 (0.92–1.15)	1.04 (0.92–1.18)	1.01 (0.90–1.13)
≥80 vs. 60–79	1.03 (0.98–1.09)	1.01 (0.96–1.07)	1.01 (0.96–1.07)
**Physical disability**	1.42 (1.35–1.49)	–	1.34 (1.27–1.41)
**Cognitive function**			
Mild vs. normal	–	1.19 (1.13–1.27)	1.20 (1.13–1.27)
Moderate vs. normal	–	1.32 (1.23–1.42)	1.31 (1.22–1.40)
Severe vs. normal	–	1.54 (1.43–1.67)	1.53 (1.43–1.64)
**Comorbidity**	1.09 (1.03–1.15)	1.16 (1.09–1.23)	–
**Never smoking**	0.93 (0.88–0.98)	0.89 (0.84–0.94)	0.91 (0.86–0.96)
**Never drinking**	0.95 (0.90–0.99)	0.96 (0.90–1.00)	0.95 (0.90–0.99)
**Exercising**	0.92 (0.88–0.96)	0.92 (0.87–0.96)	0.95(0.91–0.99)
**Ideal diet**	0.95 (0.91–0.99)	0.98 (0.93–1.04)	0.94 (0.90–0.98)
**Normal weight**	0.87 (0.83–0.91)	0.89 (0.85–0.93)	0.90 (0.96–0.94)

Supplementary Table 4. The joint effect of independent risk factors and healthy lifestyle scores on risk of mortality.

Score profile	High protecting	Median protecting	Low protecting
Low risk	1 (Ref.)	1.30 (1.19-1.42)	1.36 (1.23-1.51)
Median risk	1.53 (1.42-1.65)	1.76 (1.61-1.91)	1.83 (1.69-2.01)
High risk	2.56 (2.38-2.75)	2.82 (2.62-3.03)	2.97 (2.76-3.20)

Supplementary Table 5. Development of composite risk and protecting score and hypothetical examples.

	Factors status	Beta coefficient in Cox model[a] (β_i)	Constant Beta coefficient for one-year increase in age (β_0)	Risk points before rounding (β_i/β_0)	Integer risk points
**Hypothetical individual 1**					
Gender	Female	0	0.044	0	0
Residence	Rural	0.131	0.044	2.977	3
Physical performance	Normal	0	0.044	0	0
Cognitive function	MMSE = 23	0.293	0.044	6.649	7
Smoking status	Never smoking	−0.105	0.044	−2.394	−2
BMI	16.8 kg/m^2	0	0.044	0	0
**Hypothetical individual 2**					
Gender	Male	0.231	0.044	5.251	5
Residence	Urban	0	0.044	0	0
Physical performance	Disability	0.293	0.044	6.649	7
Cognitive function	MMSE = 28	0	0.044	0	0
Smoking status	Current smoking	0	0.044	0	0
BMI	22.5 kg/m^2	−0.128	0.044	−2.904	−3

[a]Model including age, gender, residence, ethnic, education level, economic situation, marital status, systolic BP, diastolic BP, heart rate, physical disability, cognitive function, comorbidity, smoking status, drinking, exercising, diet and BMI.
